# Immunogenicity and safety to SARS-Cov-2 vaccination in patients with systemic vasculitis

**DOI:** 10.3389/fimmu.2025.1655917

**Published:** 2025-11-17

**Authors:** Erika Biegelmeyer, Mariana de Freitas Aguiar, Priscila Dias Cardoso Ribeiro, Ketty Lysie Libardi Lira Machado, Camila Maria Paiva França Telles, Sandra Lúcia Euzébio Ribeiro, Natália Sarzi Sartori, Rodrigo Poubel Vieira de Rezende, Ana Karla Guedes de Melo, Vitor Alves Cruz, Rejane Maria Rodrigues de Abreu Vieira, Adriana Maria Kakehasi, Maria Cecília Dias Corrêa, Valderilio Feijó Azevedo, Olindo Assis Martins-Filho, Flávia Maria Matos Melo Campos Peixoto, Vanessa de Oliveira Magalhães, Maria da Penha Gomes Gouveia, Thaís Evelyn Karnopp, Katia Lino Baptista, Tâmara Santos Melo, Jozelia Rêgo, Adah Sophia Rodrigues Vieira, Anna Carolina Faria Moreira Gomes Tavares, Victória Dornelas Paz Carvalho, Vanessa Peruhype-Magalhães, Laiza Hombre Dias, Raquel Lima de Lima, Kimberly Rossana da Silva Gonçalves, Natália Rodrigues Querido Fortes, Débora Marques Veghini, Jônatas Almeida Amorim, Raiza Casian Tuão, Karina Rosemarie Lallemand Tapia, Cristiane Kayser, Charlles Heldan de Moura Castro, Maressa Barbosa Beloni Lirio, Juliana Bühring, Ricardo Machado Xavier, Andréa Teixeira-Carvalho, Viviane Angelina de Souza, Odirlei André Monticielo, Gilda Aparecida Ferreira, Marcelo de Medeiros Pinheiro, Edgard Torres dos Reis Neto, Emilia Inoue Sato, Valeria Valim, Gecilmara Salviato Pileggi, Alexandre Wagner Silva de Souza

**Affiliations:** 1 Rheumatology Division, Escola Paulista de Medicina da Universidade Federal de São Paulo (EPM-UNIFESP), São Paulo, SP, Brazil; 2 Rheumatology Division, Hospital Universitário Cassiano Antônio Moraes da Universidade Federal do Espírito Santo (HUCAM-UFES), Vitória, ES, Brazil; 3 Rheumatology Division, Faculdade de Medicina, Universidade Federal do Amazonas (FM- UFAM), Manaus, AM, Brazil; 4 Rheumatology Division, Hospital de Clínicas de Porto Alegre (HCPA), Universidade Federal do Rio Grande do Sul (UFRGS), Porto Alegre, RS, Brazil; 5 Rheumatology Division, Universidade Federal Fluminense, Niterói, RJ, Brazil; 6 Rheumatology Division, Hospital Universitário Lauro Wanderley, Universidade Federal da Paraíba (UFPB), João Pessoa, PB, Brazil; 7 Rheumatology Division, Universidade Federal de Goiás, Goiânia, GO, Brazil; 8 Rheumatology Division, Hospital Geral de Fortaleza (HGF), Universidade de Fortaleza (UNIFOR), Fortaleza, CE, Brazil; 9 Rheumatology Division, Hospital das Clínicas - Empresa Brasileira de Serviços Hospitalares (HC-EBSERH), Universidade Federal de Minas Gerais (UFMG), Belo Horizonte, MG, Brazil; 10 Rheumatology Division, Universidade Federal de Juiz de Fora, Juiz de Fora, MG, Brazil; 11 Edumed - Educação em Saúde S/S Ltda, Research Institute, Curitiba, PR, Brazil; 12 Instituto Renè Rachou, Fundação Oswaldo Cruz (FIOCRUZ-Minas), Belo Horizonte, MG, Brazil

**Keywords:** vasculitis, vaccination, COVID-19, Behçet’s disease, ANCA-associated vasculitis, Takayasu arteritis, SARS-CoV-2 vaccination

## Abstract

**Background/objectives:**

Patients with systemic vasculitis faced the risk of severe COVID-19 and high mortality during the pandemic. Although SARS-CoV-2 vaccination mitigates these outcomes, vaccine hesitancy persists, and data on immunogenicity and safety in vasculitis is still limited. This study aims to assess response to primary and booster doses of SARS-CoV-2 vaccination in systemic vasculitis.

**Methods:**

This multicenter cohort study including systemic vasculitis included patients from SAFER study (Safety and Efficacy of COVID-19 Vaccines in Rheumatic Diseases). We evaluated serum IgG levels against the SARS-CoV-2 spike protein receptor-binding domain (IgG anti-RBD) at baseline and 28 days post-vaccination, disease activity scores, new cases of COVID-19 infections, and adverse events.

**Results:**

Seventy-three patients with systemic vasculitis were included. Behçet’s disease (n=39), Takayasu arteritis (n=15), and antineutrophil cytoplasmic antibody-associated vasculitis (n=14) were the most common vasculitis forms. The majority of the patients had no comorbidities and were in remission. Seventy patients received one, 65 two, and 60 three vaccine doses. ChAdOx1 nCoV-19 (AstraZeneca/Oxford) (n=36) and CoronaVac (Sinovac) (n=25) were primarily the most common vaccines, while BNT162b2 (Pfizer–BioNTech) was usually the booster vaccine. ChAdOx1 nCoV-19 induced higher IgG anti-RBD than CoronaVac after two doses (*p*=0.002), but this difference disappeared after the booster dose. No differences in vaccine response were noted between heterologous and homologous regimens or vasculitis types. The new cases of COVID-19 (16.9%), hospitalization (1.5%), and mortality (1.5%) rates were relatively low following vaccination. Disease activity remained stable, and adverse events were mostly mild. Only one severe adverse event was observed.

**Conclusion:**

Different SARS-CoV-2 vaccines demonstrated immunogenicity and clinical effectiveness in systemic vasculitis. The three-dose schedule was safe without increasing relapse risk.

## 1 Introduction

The COVID-19 pandemic led to elevated morbidity and mortality rates in vulnerable populations, particularly immunosuppressed patients with immune-mediated rheumatic diseases (IMRDs) (1, 2). Factors such as disease activity, comorbidities, and specific medications (e.g., rituximab, cyclophosphamide, and high-dose glucocorticoids) were associated with worsening prognosis (2–7). The risk of severe COVID-19 differed among IMRDs, with worse outcomes seen in those with rheumatoid arthritis (RA), systemic sclerosis (SSc), idiopathic inflammatory myopathies, and systemic vasculitis (3, 8–11).

Systemic vasculitis is a heterogeneous group of rare, systemic autoimmune diseases characterized by inflammation and/or necrosis of blood vessel walls of varying sizes (12). The prevalence and phenotypic expression of specific forms of systemic vasculitis may vary based on ethnic and geographic factors (13–16). When compared to other IMRDs, systemic vasculitis frequently requires intensive immunosuppression due to its severity (17, 18). Furthermore, factors such as the subacute onset and protean manifestations of systemic vasculitis, delayed diagnosis, and the potentially aggressive nature of systemic vasculitis can often lead to permanent damage to target organs (17, 18). These features contribute to the increased risk of severe COVID-19 in this group of diseases (11).

Vaccination against SARS-CoV-2 is the main strategy to reduce adverse outcomes associated with COVID-19 (19–23) as evidenced by a reduction in overall population mortality following its introduction Nevertheless, vaccine hesitancy persists, usually driven by safety concerns (24–28). Although, several isolated case reports describe new-onset vasculitis following COVID-19 vaccination (29–31), large pharmacovigilance and epidemiological studies have not demonstrated a causal association, suggesting that such events may be a result of coincidental temporal clustering (32–34). In addition, we still see apprehension towards possible disease flares in patients with established systemic vasculitis after vaccination (20–34).

Numerous studies have investigated the immune response to SARS-CoV-2 vaccines in IMRDs, and most data focus on the more prevalent IMRD (35–44). Nevertheless, due to the rarity of systemic vasculitides, fewer studies have assessed immunization in this specific group of diseases (45–48). Most published studies on vasculitis immunization usually examine a single subtype of vasculitis, with relatively small sample sizes, focusing mainly on safety or immunogenicity following two or three doses of homologous vaccines (45–51). Furthermore, some IMRD cohort studies have reported lower immunogenicity in patients with vasculitis, which is usually attributed to those with AAV (52).

Studies evaluating SARS-CoV-2 vaccination in Behçet’s disease (BD) stand out for having the largest sample sizes among vasculitis patients, but all of them were conducted in Turkey, an endemic area for BD (53–55). They focused mainly on immune responses after two vaccine doses, comparing CoronaVac and BNT162b2, with additional doses assessed only for safety (53–55). Antineutrophil cytoplasmic antibody (ANCA)-associated vasculitis (AAV) is the most studied group regarding immunogenicity for SARS-CoV-2 vaccination (45–51), but most studies evaluate the vaccine response in patients under rituximab (RTX) therapy after two or three homologous doses. There is less evidence for those not receiving B-cell–depleting therapy or for those receiving heterologous schemes (45–51). In patients with giant cell arteritis (GCA), immunogenicity and safety were evaluated only up to the booster dose (56–59).

SARS-CoV-2 vaccination was less investigated in Takayasu arteritis (TAK) compared to other systemic vasculitides. Two online surveys explored the frequency of vaccination and disease relapse (28, 60). They observed higher vaccination adherence in Turkey (91%) (60), whereas coverage was lower in China (i.e., 42% received at least 2 doses) (28). Across different cohorts, vaccination was consistently well tolerated and not associated with disease flare. However, a critical knowledge gap remains. No study to date has evaluated the immunogenicity of vaccines or antibody responses in longitudinal cohorts, leaving the efficacy of SARS-CoV-2 vaccination in TAK still uncertain.SARS-CoV-2 vaccination in other forms of vasculitis, such as cryoglobulinemic vasculitis and IgA vasculitis (IgAV) in adults, were evaluated in some studies assessing immunogenicity and safety after the primary series (61–63).

Regarding safety, previous studies have shown a low frequency of relapses after two doses of vaccine in IgAV patients (63), as well as after three homologous doses in AAV and GCA (45, 64, 65). Conversely, an increased relapse rate has been reported in cryoglobulinemic and in BD after SARS-CoV-2 vaccination (54, 55, 61, 62). Although in BD most relapses were mild and predominantly mucocutaneous, severe manifestations still occurred in a few patients (55). Overall, variability across vasculitis subtypes underscores the need for more comprehensive data.

In summary, despite the growing body of evidence, key uncertainties remain. BD data are still limited to an endemic population, and most AAV studies focus on patients undergoing rituximab therapy. Furthermore, immunogenicity has not been assessed in longitudinal TAK cohorts, and the safety of additional doses of heterologous vaccine platforms remains limited for certain vasculitis subtypes. Moreover, concerns about vaccine-related relapses in clinical practice reinforce the need for studies that address both immunogenicity and safety across systemic vasculitides. Hence, this study aims to analyze the vaccine response to three doses of SARS-CoV-2 vaccines (ChAdOx1nCoV-19/Oxford–AstraZeneca, CoronaVac, and BNT162b2/Pfizer–BioNTech) in a multicenter real-life cohort of Brazilian patients with systemic vasculitis. We assessed immunogenicity, clinical effectiveness, adverse event profiles, and relapse rates. We also compared immunogenicity across vasculitis subtypes and examined the influence of csDMARDs and bDMARDs.

## 2 Materials and methods

### 2.1 Patients

This observational, multicenter, real-life, and prospective cohort study involved patients with systemic vasculitis who underwent SARS-CoV-2 vaccination between May 2021 and March 2024, across ten sites in Brazil. The study is a subset analysis of the Brazilian SAFER project (Study of Safety, Effectiveness, and Duration of Immunity after Vaccination against SARS-CoV-2 in Patients with Immune-mediated Chronic Inflammatory Diseases) (40–43). The SAFER project is supported by the Brazilian Society of Rheumatology and the Department of Science and Technology of the Ministry of Health of Brazil. Patients were eligible if they were 18 years or older, met diagnostic or classification criteria for specific forms of vasculitis (66–74) (**Supplementary Table S1**), were SARS-CoV-2 vaccination-naïve at enrollment, and had a minimum follow-up time of 4 weeks after receiving at least one dose of the SARS-CoV-2 vaccine. The following forms of systemic vasculitis were included in the study: BD, TAK, polyarteritis nodosa (PAN), IgAV, cryoglobulinemic vasculitis, primary angiitis of the central nervous system (PACNS), thromboangiitis obliterans, and AAV including granulomatosis with polyangiitis (GPA), and eosinophilic granulomatosis with polyangiitis (EGPA).

Exclusion criteria included pregnancy, history of severe adverse reactions to any previously administered vaccines, and secondary causes of immunosuppression such as living with HIV (i.e., CD4^+^ T cell count <200 cells/mm3), organ transplant, primary immunodeficiency, cancer, or history of disorders of the thymus (e.g., myasthenia gravis, thymoma, absence of the thymus or surgical removal).SARS-CoV-2 vaccination was postponed for patients who had received rituximab within the last six months, intravenous (IV) cyclophosphamide pulse therapy within the last three months, IV glucocorticoid (GC) pulse therapy, IV immunoglobulins, or underwent plasmapheresis within the previous 30 days, as well as those who received any blood product transfusions within 30 days before study inclusion. Additionally, vaccination was postponed for at least four weeks after suspicion or confirmed diagnosis of SARS-CoV-2 (i.e., via RT-PCR or rapid test), or two weeks after receiving another type of vaccine.

This study was performed according to Helsinki’s declaration and its updates. The institutional review board approved the study protocol at each site and all study participants gave written informed consent (CAAE 43479221.0.1001.5505).

### 2.2 Vaccines

The following SARS-CoV-2 vaccines were included in the analysis: the inactivated SARS-CoV-2 virus vaccine (CoronaVac/Sinovac/Butantan), the mRNA-based vaccine BNT162b2 (Pfizer-BioNTech), the adenoviral vector vaccines ChAdOx1 nCoV-19 (AstraZeneca/Oxford), and Ad26COV2-S (Janssen/Johnson&Johnson). This study was conducted in accordance with the protocols outlined by Brazil’s National Immunization Program, and vaccines were made available by the Brazilian public health system (75, 76). CoronaVac was administered in two doses 28 days apart; BNT162b2 in two doses 21 days apart; ChAdOx1 in two doses 12 weeks apart; and Ad26.COV2-S as a single-dose scheme. Booster doses were recommended at least four months after completion of the primary series or two months for Ad26.COV2-S.

### 2.3 Follow-up assessments

The study visit schedule included a baseline visit (T0) before vaccination, and three follow-up visits (i.e., T1, T2, and T3), carried out at least 28 days after the administration of each vaccine dose, totaling three doses. The T1 visit occurred after the first dose, T2 after the completion of the full vaccination schedule, and T3 after the booster dose. During each visit, we collected blood samples and assessed patients for signs and symptoms related to vasculitis using specific disease activity tools for each form of vasculitis, as well as therapeutic interventions. A diary of symptoms was provided for patients to complete for 28 days after vaccination, and active monitoring of severe adverse events was conducted at each subsequent visit. The data were recorded using the Research Electronic Data Capture (REDCap) tool.

### 2.4 SARS-CoV-2 serologic assays

Immunogenicity was assessed by measuring IgG antibodies against the SARS-CoV-2 spike receptor-binding domain (IgG-RBD) using a chemiluminescent microparticle immunoassay (CMIA) for qualitative and semi-quantitative detection (SARS-CoV-2 IgG-II Quant assay, Abbott Laboratories, Green Oaks, IL, USA) (77). The titers of IgG-RBD antibodies were expressed as geometric mean (GMT) and described in binding antibody units (BAU/mL). Seropositivity was defined as IgG-RBD antibody titers of 7.1 BAU/mL or higher. The increase in IgG-RBD GMT titers after each vaccine dose was compared between different doses during the follow-up and among different vaccine types. The rate of IgG-RBD titer increase after each dose was calculated.

### 2.5 Diagnostic confirmation of COVID-19 infection and suspected cases

Confirmed cases of COVID-19 were defined as patients testing positive for SARS-CoV-2 via reverse transcription-polymerase chain reaction (RT-PCR) or validated antigen tests. Due to limited testing accessibility in our population, suspected cases were also included to minimize data loss. Suspected cases were classified according to Brazilian Ministry of Health definition, as patients presenting characteristic COVID-19 symptoms, including fever, dry cough, and respiratory distress, in conjunction with a loss of smell or taste or a history of close contact with a confirmed COVID-19 case within the preceding two weeks.

### 2.6 Tools to assess disease activity

Disease activity was assessed using specific tools for each type of vasculitis. The Birmingham Vasculitis Activity Score (BVAS) v3 was used to evaluate AAV, PAN, cryoglobulinemic vasculitis, and IgAV patients. Active disease was defined as BVAS v3 ≥ 1 (78). The short form of the Brazilian version of Behçet’s Disease Current Activity Form (BR-BDCAF) was used to evaluate disease activity in BD, and a score ≥ 2 was regarded as an active disease (79). In TAK patients, disease activity was defined according to Kerr’s criteria (80).

### 2.7 Study endpoints

The primary endpoint of this study was the immunogenicity of SARS-CoV-2 vaccination after the booster dose, evaluated as IgG-RBD GMT titers and seropositivity four weeks following the booster dose. Secondary endpoints included seropositivity after each dose, and the comparison of vaccine responses among different SARS-CoV-2 vaccines, as well as between homologous and heterologous vaccination schemes, and among different types of vasculitis. Additionally, the influence of current therapy on the immunogenicity of SARS-CoV-2 vaccination was assessed, as well as the clinical effectiveness of SARS-CoV-2 vaccines and vaccination schemes during the follow-up period. Safety outcomes included the number of disease relapses, changes in disease activity scores after each dose, and the adverse events profile after the SARS-CoV-2 vaccination.

### 2.8 Statistics

The proportions between groups were compared using the chi-square test or Fisher’s exact test for categorical variables. For continuous variables, the mean and standard deviation (SD), as well as the median and interquartile range (IQR), were calculated according to the normality of the data. The interquartile range was expressed as Q1–Q3. Continuous variables were compared using the Student’s t-test or the Mann-Whitney test, respectively. For comparisons among three or more groups, one-way analysis of variance (ANOVA) or the Kruskal-Wallis test was used.

For the longitudinal analysis of IgG titers, data were normalized using base 10 logarithms, and the median increase in titers was calculated after each dose. The variation in normalized IgG titers over time was assessed using repeated measures (ANOVA). The rate of increase between doses was expressed as medians and compared using the non-parametric Wilcoxon/Mann-Whitney test, with Bonferroni correction.

To identify predictors of higher or lower anti-RBD IgG titer responses after the booster dose, univariate linear regression was used to select variables for the multivariate analysis. A p-value < 0.2 was the criterion for the inclusion of an independent variable in the backward stepwise multivariate model. If this criterion was not met, a biological model was constructed for multivariate linear regression analysis, including the main factors known to influence vaccine responses. All statistical analyses were carried out using the Stata statistical package (v.17) and R (v.4.2.0).

## 3 Results

### 3.1 Profile of the whole cohort

**Figure 1:**
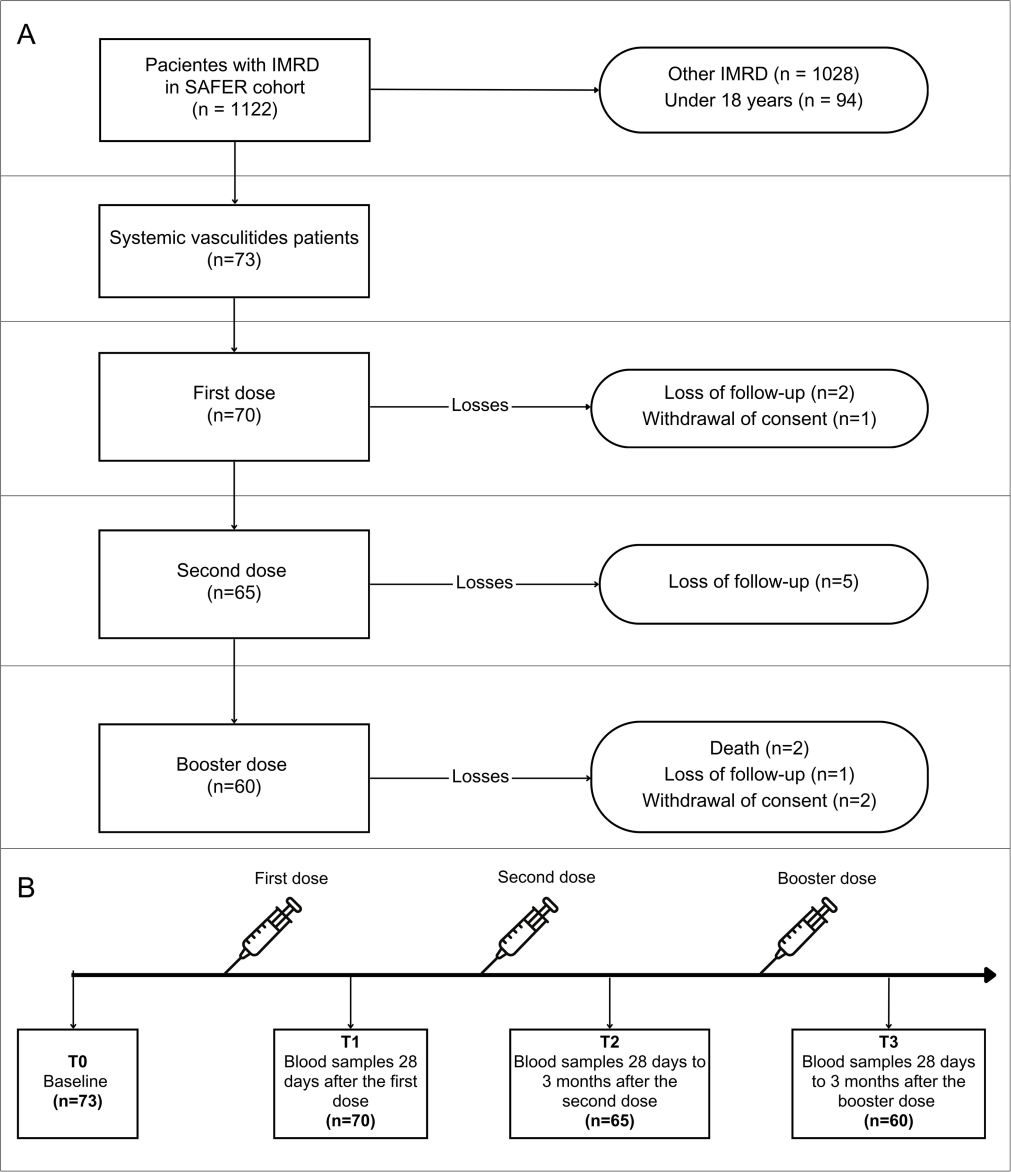
Flow chart and follow-up reporting the number of patients investigated for Sars-Cov-2 immunogenicity at different time points in the study. **(A)** shows the patient inclusion flowchart; **(B)** illustrates the study follow-up schedule of visit intervals concerning vaccine doses. IMRD, Immune-mediated rheumatic diseases; SAFER, Study on Safety, Effectiveness, and Duration of Immunity after SARS-CoV-2 Vaccination in Patients with Chronic Immune-Mediated Inflammatory Diseases.

Seventy-three patients with systemic vasculitis were assessed at baseline, 70 patients received the first SARS-CoV-2 vaccine dose, 65 completed the primary vaccination series, and 60 patients received the booster dose (**Figure 1**).

**Figure 2:**
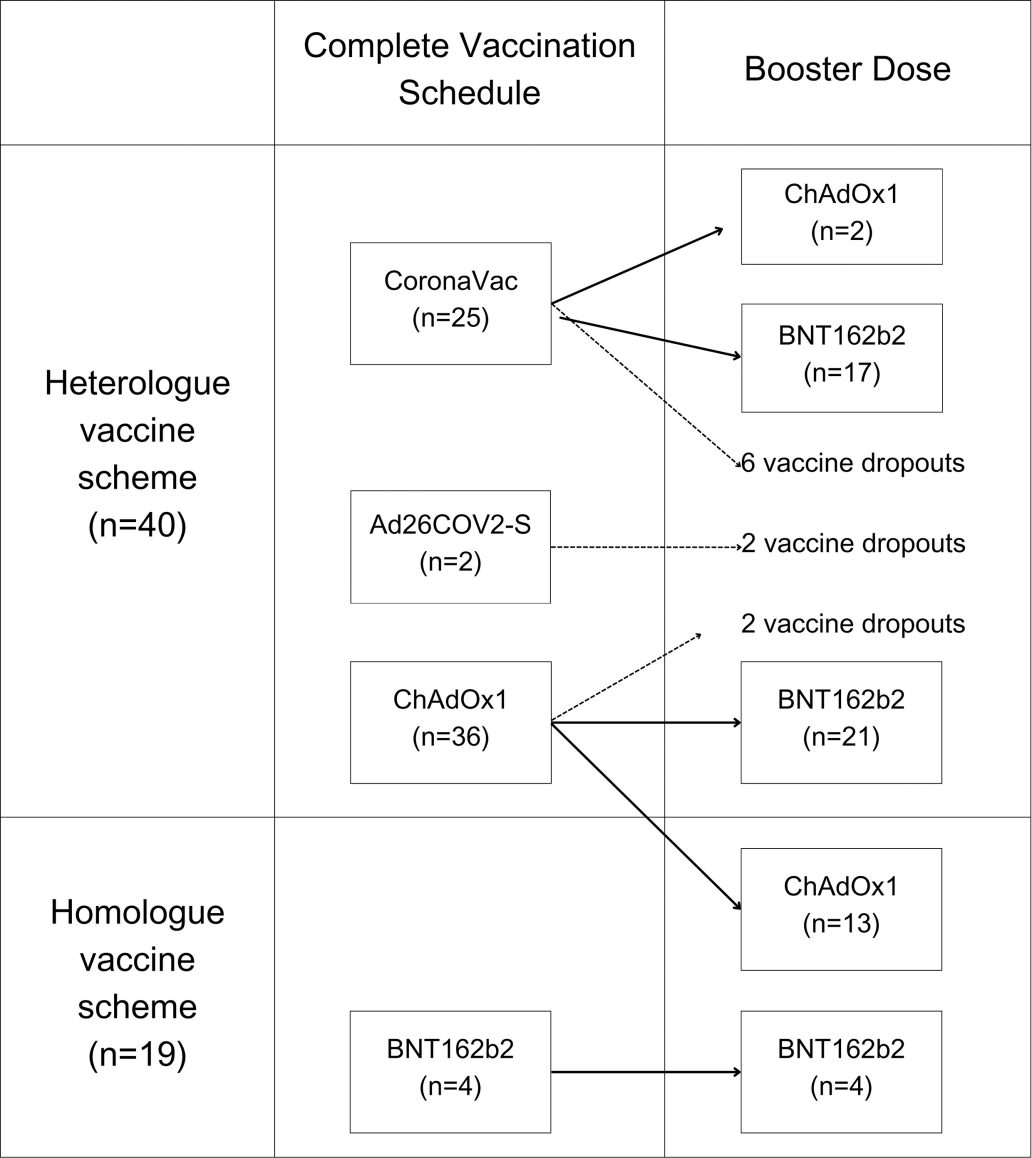
Vaccination distribution between homologous and heterologous vaccination schedules.

At baseline, the majority of patients were female, Whites and Mestizos represented the largest group. **Table 1** depicts demographic parameters, and the frequency of each vasculitis form and its therapy. The three main vasculitis forms included in the study were BD, TAK, and AAV. About half of the cohort had comorbidities, with hypertension and obesity being the most common. In terms of therapy, over half of the patients were taking csDMARDs, and more than one-third were on bDMARDs, primarily TNFi or tocilizumab. Only a few were under rituximab therapy. Glucocorticoids were used by about one-third of the patients, typically in low daily doses. Approximately half of the patients had at least one comorbidity, including hypertension (31.5%), obesity (12.3%), diabetes (8.2%), heart disease (4.1%), and lung disease (1.4%). None of the patients had end-stage kidney disease. Around two-thirds of participants underwent heterologous vaccination regimens combining ChAdOx1 nCoV-19, CoronaVac, and BNT162b2, whereas homologous regimens were mainly based on ChAdOx1 nCoV-19 (**Figure 2**).

**Table 1:** Baseline features of vasculitis patients undergoing different SARS-CoV-2 vaccination schemes in the cohort.

Variables	CoronaVac (n = 25)	ChAdOx1 nCoV-19 (n = 36)	*p*	Heterologous scheme (n = 40)	Homologous scheme (n 19)	*p*
Females, n (%)	19 (76.0)	23 (63.9)	0.32	31 (77.5)	10 (52.6)	0.053
Age, years	34.4 ±10.7	39.9 ±11.5	0.067	38.9 ±11.0	38.1 ±11.5	0.79
Race,						
Whites, n (%)	12 (48.0)	18 (50.0)		22 (55.0)	12 (63.2)	0.69
Blacks, n (%)	4 (16.0)	4 (11.1)	0.87	3 (7.5)	2 (10.5)	
Mestizos, n (%)	9 (36.0)	14 (38.9)		15 (37.50)	5 (26.3)	
BMI, kg/m2,	27.6 ± 6.3	26.5 ± 4.7	0.46	27.5 ± 5.0	26.5 ± 7.7	0.56
No comorbidities, n (%)	11 (44.0)	19 (52.8)	0.50	19 (47.5)	10 (52.6)	0.71
Pre-exposed to COVID-19, n (%)	5 (20.0)	1 (2.8)	0.038*	4 (10.0)	1 (5.3)	1.000
Systemic vasculitis						
AVV, n (%)	6/14 (42.8)	8/14 (57.2)	0.87	11/12 (91.6)	1/12 (83.4)	0.04*
BD, n (%)	9/24 (37.5)	15/24 (62.5)	0.65	16/26 (61.5)	10/26 (38.5)	0.36
TAK, n (%)	5/11 (45.4)	7/11 (63.6)	0.95	8/11 (72.7)	3/11 (27.3)	0.69
Oral GCs, n (%)	11 (44.0)	12 (33.3)	0.43	15 (37.5)	6 (31.6)	0.77
Up to 5mg/day	8/23 (34.8)	5/12 (41.7)	NA	6/15 (40.0)	1/6 (16.7)	NA
≥6 a 10 mg/day, n (%)	5/23 (21.7)	4/12 (33.3)	NA	2/15 (13.3)	4/6 (66.7)	NA
≥11 a 20 mg/day, n (%)	6/23 (26.1)	2/12 (16.7)	NA	4/15 (26.7)	0/6 (0.0)	NA
>20 mg/day, n (%)	4/23 (17.4)	1/12 (8.3)	NA	3/15 (20.0)	1/6 (16.7)	NA
csDMARD, %	12 (48.0)	22 (61.1)	0.31	23 (57.5)	9 (47.4)	0.46
Methotrexate ≤20mg/week, n (%)	0/2 (0.0)	5/6 (83.3)	NA	3/6 (50.0)	2/2 (100.0)	NA
Methotrexate >20mg/week, n (%)	2/2 (100.0)	1/6 (16.7)	NA	3/6 (50.0)	0/2 (0.0)	NA
Mycophenolate mofetil, n (%)	2/25 (8.0)	6/36 (16.7)	0.45	7/40 (17.5)	1/19 (5.3)	0.42
Hydroxychloroquine, n (%)	0/25 (0.0)	1/36 (2.8)	1.00	0/40 (0.0)	1/19 (5.3)	0.32
bDMARD						
TNFi or tocilizumab, n (%)	4/25 (16.0)	13/36 (36.1)	0.15	11 (27.5)	9 (47.4)	0.15
Rituximab, n (%)	2/25 (8.0)	2/36 (5.6)	1.00	4/40 (10.0)	0/19 (0.0)	0.29

AAV, ANCA associated Vasculitis; BD, Behçet’s disease; bDMARD, Biological disease modifying antirheumatic drugs; BMI, Body mass index; csDMARD, Conventional synthetic disease modifying antirheumatic drugs; GC, Glucocorticoid; n, Number of patients; NA, Not applicable; TAK, Takayasu arteritis; TNFi, Tumor necrosis factor inhibitors; * - Flags significant results.

### 3.2 Comparison of baseline features of study participants

Vasculitis patients originally immunized by CoronaVac had a higher frequency of previous COVID-19 infection compared to those vaccinated with ChAdOx1 nCoV-19. Additionally, AAV patients were more frequently vaccinated with a heterologous regimen than with a homologous one. These differences, along with the frequency of bDMARD use (e.g., TNFi/tocilizumab in BD and TAK; rituximab in a few AAV patients), are summarized in **Table 1** and **Supplementary Table S1**. No other significant differences regarding demographic data, comorbidities, diagnosis of vasculitis, or therapy at the baseline visit were found between the different SARS-CoV-2 vaccination schemes.

### 3.3 Immunogenicity to SARS-CoV-2 vaccines

**Figure 3:**
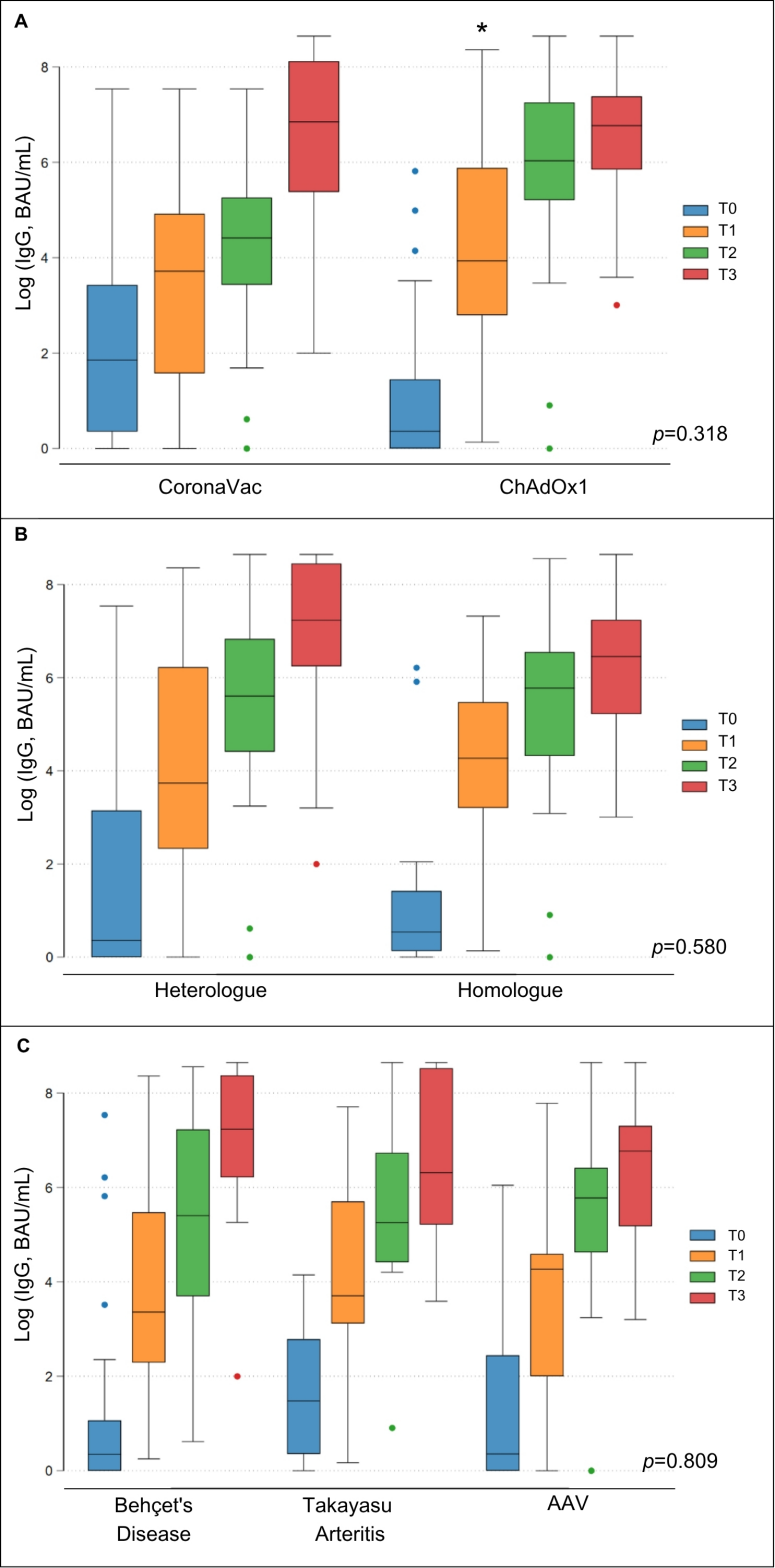
Immunogenicity of SARS-CoV-2 vaccination in subgroups of vasculitis patients. Boxplot graphs describe the comparison of the geometric means of IgG anti-RBD antibodies between vasculitis patients vaccinated with Coronavac or ChAdOx1 nCoV-19 **(A)**, between those immunized with heterologous or homologous vaccine schemes **(B)** and between different forms of vasculitis **(C)**. T0, baseline visit; T1, 28 days after first dose; T2, 28 days after second dose; T3, 28 days after third dose of SARS-CoV-2 vaccine; *Flags significant results.

**Figure 4:**
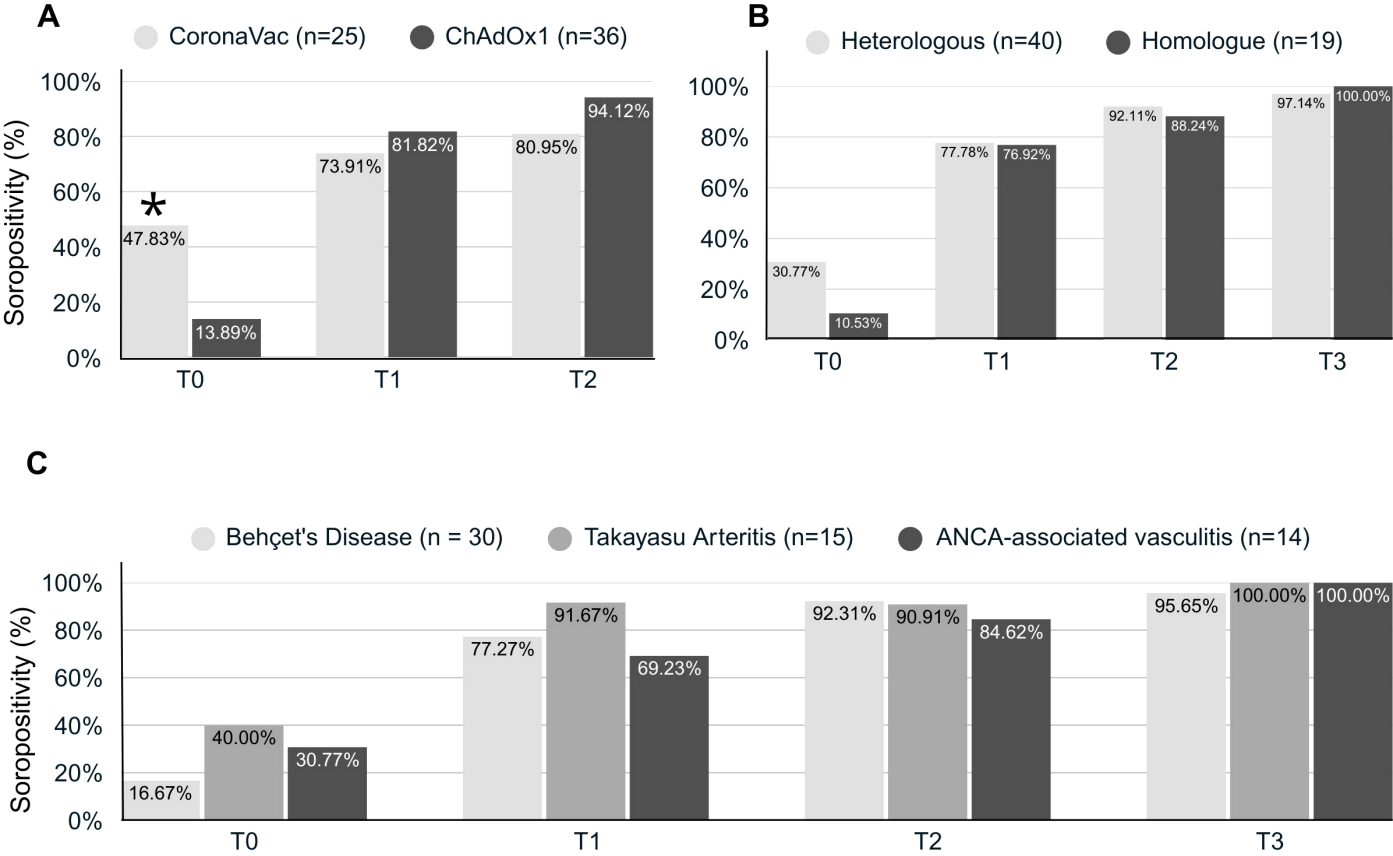
Seropositivity against SARS-CoV-2 in vasculitis patients. Bar charts graphs describe the comparison of the seropositivity (responder) of IgG anti-RBD antibodies between vasculitis patients vaccinated with Coronavac or ChAdOx1 nCoV-19 **(A)**, between those immunized with heterologous or homologous vaccine schemes **(B)** and between different forms of vasculitis **(C)**. T0, baseline visit; T1, 28 days after first dose; T2, 28 days after second dose; T3, 28 days after third dose of SARS-CoV-2 vaccine; *Flags significant results.

At the baseline visit before SARS-CoV-2 vaccination, the CoronaVac group had higher mean levels of IgG-RBD titers (**Supplementary Table S2**, **Figure 3A**) and a higher seropositivity rate (47.8% vs. 13.9%; *p*=0.004) compared to the ChAdOx1 nCoV-19 group (**Figure 4A**, **Supplementary Table S3**). These findings are consistent with a higher COVID-19 pre-exposure rate in the CoronaVac group. After the primary SARS-CoV-2 vaccination series (i.e., T2 visit), the ChAdOx1 nCoV-19 group achieved significantly higher levels of immunogenicity compared to the CoronaVac group (**Supplementary Table S2**, **Figure 3A**), even though there was no significant difference regarding the seropositivity rate between the groups (94.1% vs. 90.9% respectively; *p*=0.13) (**Figure 4A**). After the booster dose (i.e., T3 visit), both groups achieved similar IgG-RBD titers, regardless of the vaccine scheme used (**Supplementary Table S2**).

When comparing heterologous and homologous SARS-CoV-2 vaccination schemes, no significant differences were observed in the mean IgG anti-RBD titers between both schemes (*p*=0.580). However, the heterologous group had only a tendency for higher mean IgG anti-RBD titers (*p*=0.073) at the T3 visit (**Supplementary Table S2**, **Figure 3B**). The seropositivity rates were also similar between patients who underwent a homologous and heterologous SARS-CoV-2 vaccination scheme at baseline and during the follow-up (*p* > 0.05) (**Figure 4B**, **Supplementary Table S3**).

When patients with BD, TAK, and AAV were compared regarding levels of immunogenicity to SARS-CoV-2 vaccination, no significant differences were found in mean levels of IgG-RBD antibodies during the follow-up period (*p*=0.809) (**Supplementary Table S2**, **Figures 3C**, **4C**, **Supplementary Table S4**). A significant rise in IgG-RBD antibody titers was observed within each group over time (*p*<0.0001).

For all groups, the IgG-RBD GMT increment rate was higher after the first dose of the SARS-CoV-2 vaccine, but this IgG-RBD GMT rise gradually declined with subsequent vaccine doses (**Table 2**). An exception for this was observed in the group initially allocated to vaccination with CoronaVac, as this group exhibited the lowest IgG-RBD GMT increment after the first and particularly after the second dose. Interestingly, this group showed higher GMT increments after the booster dose, typically administered with BNT162b2, catching up with IgG-RBD GMT levels comparable to levels achieved by other vaccines.

**Table 2:** Median of the log increment rates of anti-RBD IgG levels after each vaccine dose.

Interval between timepoints	CoronaVac (n=25)	ChAdOx1 nCoV-19 (n=36)	*p*	Heterologous (n=40)	Homologous (n=10)	*p*
T0 - T1	54.84 (12.40-158.05)	222.76 (116.57-831.40)	0.007*	145.51 (35.53-426.04)	257.97 (116.57-768.74)	0.62
T1 - T2	1.46 (-4.06-50.93)	46.61 (5.82-97.15)	0.099*	33.97 (-1.51-91.30)	28.49 (0.22-72.04)	0.89
T2 - T3	30.52 (-1.03-90.67)	3.20 (-8.58-35.48)	0.14	15.21 (-1.22-50.57)	4.08 (-1.19-51.88)	0.96
*p*	0.079	<0.001*		<0.001*	0.023*	

T0, Baseline; T1, 28 days after the 1st dose; T2, 28 days after the 2nd dose; T3, 28 days or more after the 3rd dose; *significant results. Continuous variables are presented as medians (Q1-Q3).

### 3.4 Clinical effectiveness SARS-CoV-2 vaccination in vasculitis

After the first two doses of SARS-CoV-2 vaccination, three patients (4.9%) developed either suspected or confirmed COVID-19, all of whom were in the CoronaVac group (**Supplementary Table S5**). Following the booster dose, ten patients (16.9%) developed COVID-19, but no significant differences were found between the different vaccine groups (**Supplementary Table S6**).

Only one case of severe COVID-19 that resulted in death was observed in the study. The patient was a 63-year-old and had IgAV with renal involvement characterized by nephrotic-range proteinuria, with chronic kidney disease (CKD) as a permanent damage. The patient had been treated with RTX one year prior to vaccination and had been maintained on long-term prednisone therapy between 6–10 mg/day for approximately ten years. Despite remission, disease recurred upon discontinuation of low-dose prednisone. After vaccination, the patient did not show any humoral response following the initial two doses of ChAdOx1 nCoV-19. The first reactive serology (IgG anti-RBD: 2.2 log10 BAU/mL) was detected only after the booster dose. Severe COVID-19 symptoms emerged two months after the booster, leading to ICU admission due to respiratory failure, and ultimately resulting in death. The adverse outcome was attributed to multifactorial risks, including CKD, advanced age, and vaccine anergy related to prior RTX and chronic GC therapy. Importantly, no IgAV disease activity was detected during this adverse event. In this cohort, no other severe cases of COVID-19 were reported. Thus, hospitalization and mortality rates were 1.5% each among 65 patients who received three vaccine doses.

### 3.5 Predictors of IgG anti-RBD antibody titers after vaccination

The univariate linear regression was first used as a screening tool to explore potential predictors of anti-RBD IgG titers after the third SARS-CoV-2 vaccine dose. Although only one variable met the conventional p-value threshold of <0.2, we decided to build a multivariate model including key variables of clinical relevance (i.e., rituximab, immunosuppressants, type of vasculitis, and vaccination scheme), regardless of their univariate statistical significance. This approach aimed to account for potential confounders while minimizing model overfitting. The analysis showed that the immune response was not significantly affected by medication use, vaccination schedule, or type of vasculitis in this study (**Table 3**).

**Table 3:** Univariate and multivariate linear regression to evaluate predictors of immunogenicity response after three doses of vaccine.

IgG anti-RBD titers after the 3^rd^ dose	Univariate				Multivariate			
	Unstandardized β coefficient	[95% CI]		*P*	Unstandardized β coefficient	[95% CI]		*P*
Gender								
Male	0	–		–	0	–		–
Female	0.211	-0.717	1.139	0.650	-0.294	-1.558	0.970	0.638
Age in years	0.007	-0.034	0.048	0.751	0.014	-0.048	0.076	0.652
Rituximab	0.257	-1.662	2.176	0.789	0.626	-2.340	3.592	0.670
TNFi and Tocilizumab	0.327	-0.649	1.302	0.505	0.145	-1.195	1.484	0.827
csDMARDs	0.119	-0.860	1.098	0.808	-0.142	-1.525	1.240	0.835
Glucocorticoids	-0.542	-1.427	0.344	0.225	-0.160	-1.451	1.131	0.802
Vasculitis subtype								
Behçet	0	–		–	0	–		–
Takayasu	-0.385	-1.621	0.851	0.533	-0.415	-1.950	1.120	0.585
AAV	-0.432	-1.628	0.764	0.470	-0.927	-2.589	0.734	0.264
Vaccination Schedule								
Heterologous	0	–		–	0	–		–
Homologous	-0.863	-1.810	0.084	0.073	-0.989	-2.314	0.336	0.138

95% CI, 95% confidence interval; AAV, ANCA-associated vasculitis; csDMARD, Conventional synthetic disease modifying antirheumatic drugs; RBD, Receptor binding domain.

### 3.6 Safety of SARS-CoV-2 vaccination in vasculitis patients

The frequency of patients presenting active disease was similar at inclusion and during follow-up with successive vaccine doses (**Table 4**). The BVAS v3 and BR-BDCAFs scores did not change significantly before or after SARS-CoV-2 vaccination, as the frequency of patients in remission and those remaining at very low disease activity remained stable throughout the study (**Table 4**). No thromboembolic events were reported in BD patients after SARS-CoV-2 vaccination. However, we observed one severe adverse event related to a life-threatening disease relapse in an 80-year-old female patient with EGPA. Before receiving the booster dose, the patient had signs of mild active disease, and approximately five days after the BNT162b2 booster, the disease activity flared up with the development of myocarditis, fleeting pulmonary infiltrates, and eosinophilia. The possibility of myocarditis associated with the BNT162b2 vaccine was ruled out, as the patient did not fit the typical profile of individuals who develop this vaccine-related complication and had other features consistent with active EGPA. The patient was hospitalized and treated with intravenous methylprednisolone and cyclophosphamide pulse therapy, as well as with high-dose glucocorticoids, showing a good response to therapy. In the investigator’s opinion, immunization was likely to contribute to the vasculitis flare-up of an underlying disease activity.

**Table 4:** Assessment of disease activity in ANCA-associated vasculitis and Behçet’s disease before and after each vaccine dose.

Vasculitis	T0	T1	T2	T3
Disease activity scores				
AAV	2.50 (2.00-3.00)	3.00 (2.00-4.00)	3.00 (2.00-4.00)	4.00 (4.00-5.00)
Behçet’s Disease	2.00 (2.00-2.50)	2.50 (2.00-3.00)	2.00 (2.00-2.50)	2.00 (2.00-3.00)
Active disease				
AAV, n (%)	4/12 (33.3)	0/8 (0.0)	2/12 (16.7)	2/12 (16.7)
Behçet’s disease, n (%)	8/23 (34.7)	2/11 (18.2)	4/25 (16.0)	2/23 (8.7)
Takayasu arteritis, n (%)	2/11 (18.2)	1/6 (16.7)	2/12 (16.7)	1/11 (9.1)

n, Number of patients; AAV, antineutrophil cytoplasmic antibody-associated vasculitis; T0, Baseline; T1, 28 days after the 1st dose; T2, 28 days after the 2nd dose; T3, 28 days or more after the 3rd dose; *Flags significant results; results are presented as median and interquartile range (Q1-Q3) of patients presenting scores ≥ 1 and ≥ 2 for Birmingham Vasculitis Activity Score version 3 and the Brazilian simplified version of the Behçet’s Disease Current Activity Form, respectively.

Most adverse events (AEs) related to SARS-CoV-2 vaccination were mild. Injection-site pain and skin rashes were more frequent in vasculitis patients undergoing the first ChAdOx1 nCoV-19 dose compared to CoronaVac. There were no other significant differences regarding AEs between vaccination groups, neither in the complete schedule (**Table 5**) nor in the booster dose scheme (**Supplementary Table S7**).

**Table 5:** Comparisons of safety between CoronaVac and ChAdOx1 nCoV-19 vaccines in vasculitis patients after the complete vaccination schedule.

Adverse events	CoronaVac (n = 25)	ChAdOx1 nCoV-19 (n = 36)	*p*
Up to 28 days after the first dose			
Site-injection pain, n (%)	9/21 (42.9)	30/36 (83.3)	0.002*
Skin rashes, n (%)	0/21 (0.0)	7/36 (19.4)	0.039*
Nausea or vomiting, n (%)	5/21 (23.8)	12/36 (33.3)	0.55
Fatigue, n (%)	7/21 (33.3)	18/36 (50.0)	0.22
Headache, n (%)	9/21 (42.9)	14/36 (38.9)	0.77
Myalgia, n (%)	5/21 (23.8)	13/36 (36.1)	0.39
Arthralgia, n (%)	5/21 (23.8)	12/36 (33.3)	0.55
Fever, n (%)	4/21 (19.1)	8/36 (22.2)	1.00
Dizziness, n (%)	6/21 (28.6)	8/36 (22.2)	0.59
Up to 28 days after the second dose			
Site-injection pain, n (%)	5/19 (26.3)	14/33 (42.4)	0.37
Skin rashes, n (%)	0/19 (0.0)	3/33 (9.1)	0.29
Nausea or vomiting, n (%)	2/19 (10.5)	7/33 (21.2)	0.46
Fatigue, n (%)	6/19 (31.6)	10/33 (30.3)	0.92
Headache, n (%)	3/19 (15.8)	9/33 (27.3)	0.50
Myalgia, n (%)	5/19 (26.3)	8/33 (24.2)	1.00
Arthralgia, n (%)	3/19 (15.8)	10/33 (30.3)	0.33
Fever, n (%)	1/19 (5.3)	5/33 (15.2)	0.40
Dizziness, n (%)	3/19 (15.8)	6/33 (18.2)	1.00

N, Number of patients; *Flags significant results.

A total of four SAEs (serious adverse events) were reported, two of which were described above (i.e., severe COVID-19 in a patient with IgAV and myocarditis due to EGPA). In addition to these events, there was one death following severe dengue infection in a patient with BD, unrelated to SARS-CoV-2 vaccination, and one case of intracranial hemorrhage due to the rupture of an intracranial aneurysm in a patient with BD on TNFi therapy, who was in remission. This severe event occurred several months after the booster dose and is unlikely to be related to SARS-CoV-2 vaccination. Only one of the severe adverse events observed in this study was directly related to the SARS-CoV-2 vaccine, that is, myocarditis due to EGPA in a patient who received the vaccination while presenting with mild active disease.

## 4 Discussion

In this prospective cohort study of planned vaccination, we evaluated the immune vaccine response, clinical effectiveness, and safety of SARS-CoV-2 vaccination in patients with systemic vasculitis. All vaccine schedules evaluated in this study demonstrated an increase in anti-RBD IgG titers, with the ChAdOx1 nCoV-19 vaccine showing greater immunogenicity than the CoronaVac vaccine after the complete schedule. However, this initial difference in vaccine response between ChAdOx1 nCoV-19 and CoronaVac disappeared after the booster dose, usually done with the BNT162b2 vaccine. Additionally, there were no differences in the immune response between homologous and heterologous vaccine schedules. The rate of suspected or confirmed COVID-19 cases after the booster dose was as low as 16.9%, accompanied by a low COVID-19 mortality (i.e., 1.5%). The use of immunosuppressive medications was not shown to affect the immunogenicity of SARS-CoV-2 vaccination in this population. However, we acknowledge that this study may not have sufficient statistical power to detect associations. In terms of safety, all SARS-CoV-2 vaccines demonstrated a favorable safety profile as no increase in disease relapses was observed throughout the study, and most reported adverse events were mild in intensity. However, there was one serious adverse event attributed to vaccination, occurring in a patient with active disease at the time of immunization.

The ChAdOx1 nCoV-19 vaccine induced higher mean GMT anti-RBD IgG titers and a greater increment rate than CoronaVac after the complete schedule. This finding is consistent with previous studies that identified lower immunogenicity of inactivated vaccines compared to other vaccine platforms (53, 81). However, the group vaccinated with CoronaVac had higher pre-existing viral exposure, which may have influenced the lower increment rate to vaccination observed in this group. Indeed, it is well-known that a short interval between SARS-CoV-2 infection and vaccination can result in a reduced immune response (82, 83). Despite these initial differences, seropositivity remained similar between both vaccines throughout the primary vaccination schedule.

The booster dose increased anti-RBD IgG titers in all patient groups, regardless of the initially administered vaccine platforms. It proved to be particularly important in patients with a less robust immunogenic response to the first two SARS-CoV-2 vaccine doses. A previous study including Brazilian AAV patients showed that a booster dose of CoronaVac increased antibody titers as well, indicating the benefits of the booster even with an inactivated vaccine (47). On the other hand, other studies indicate that some patients persisted unresponsive even after receiving the third dose of mRNA vaccines (45), suggesting that additional SARS-CoV-2 vaccine doses are likely needed for adequate protection in non-responders. Furthermore, the booster dose provided extra protection against mutant strains of SARS-CoV-2, such as Delta and Omicron, across various populations (45, 84).

Our population did not differ in immunogenicity between homologous and heterologous vaccine schedules. This topic has generated controversy in literature. While some studies found superiority in heterologous schedules (42, 81, 85, 86), other - including ours- did not (87), and some even favored homologous boosting (88). We believe that this variability in results of immunogenicity between homologous and heterologous vaccine schedules may be attributed to the specific characteristics of each studied population and size of the sample. It is possible that diversifying vaccine platforms does not significantly impact the immune response as long as the vaccines used are effective and administered in a schedule of at least three doses.

Our study demonstrated a low frequency of suspected or confirmed COVID-19 (16.9%) following the complete vaccination schedule and booster dose. Another study including BD patients reported 10.1% COVID-19 cases after two doses of CoronaVac and 1.4% after BNT162b2, when patients were still under social distancing measures (54). In our study, the booster dose was close to the end of social distancing measures, accompanied by modifications in population social behavior and the emergence of the Delta and Omicron SARS-CoV-2 variants. This suggests that our patients with vasculitis may have had a higher viral exposure to SARS-CoV-2 during the booster phase compared to the primary series.

We also report low hospitalization (1.5%) and mortality (1.5%) rates due to COVID-19. For comparison reasons, the hospitalization rate of COVID-19 in Latin America prior to SARS-CoV-2 vaccination was 22%, with a mortality rate of 4% (89). For individuals living with vasculitis, these rates were even higher, ranging between 23% and 38% for hospitalizations and between 9% and 28% for mortality (8–11). Other studies including IMRD patients have demonstrated similar benefits of SARS-CoV-2 vaccine effectiveness, showing a reduction in hospitalizations from 25.0% to 4.8% and mortality from 5.7% to 0% in patients with a complete vaccination schedule (41, 65, 90). These studies found that COVID-19 mortality was associated with high disease activity and the use of GC, while the number of SARS-CoV-2 vaccine doses was shown to be a protective factor. To the best of our knowledge, our study is the first to demonstrate low mortality and hospitalization rates in patients with systemic vasculitis after SARS-CoV-2 vaccination. It is worth noting that the only severe case of COVID-19 occurred in a patient who did not adequately respond to the initial SARS-CoV-2 vaccine doses, reinforcing the importance of booster doses for protection against severe COVID-19 outcomes.

Robust evidence supports that immunosuppressive therapies such as GCs, MTX ≥20 mg, MMF, and RTX are major determinants of impaired SARS-CoV-2 vaccine immunogenicity (50, 58, 91–94). In GCA, for example, patients under MTX therapy have shown reduced humoral responses after two doses of the SARS-CoV-2 vaccine, with recovery only after receiving booster doses. In contrast, patients treated with tocilizumab mounted a higher antibody response (57–59). This effect was even more pronounced when MTX was combined with moderate to high doses of GC (58). In AAV, RTX-treated patients consistently exhibited lower seropositivity rates and faster antibody waning compared to non-RTX patients, even after a booster dose (48, 49). Nevertheless, SARS-CoV-2 vaccination can still induce cellular responses in RTX users, and provide partial protection, despite B-cell depletion (48, 49). In studies including BD patients, TNFi was associated with lower serological responses after one dose of CoronaVac. However, it did not affect by mRNA vaccine immunogenicity (53).

On the other hand, in our cohort, the univariate and multivariate analyses did not show that these medications significantly affected on SARS-CoV-2 vaccine immunogenicity. We acknowledge that this discrepancy is likely due to the limited number of patients taking these medications and the timing of vaccination in relation to treatment. Only five patients were on RTX, and they were all vaccinated at least six months after their last infusion, a period when B-cell repopulation is likely to occur in most patients. Regarding GCs, most of the literature describes reduced responses after the first or second dose. However, a third dose may compensate for the initial deficit (57–59). Moreover, only a few patients were taking MMF (n=8) or high-dose MTX (n=3), which limited the possibility of analyzing these drugs individually. Therefore, these findings should not be interpreted as evidence that immunosuppressive therapy has no impact on vaccine responses. Rather, they should be understood as a consequence of the small number of patients taking these drugs in our cohort, the distribution of treatments, and the possible compensatory effect of booster doses (45, 46, 48, 49, 53, 56–59).

Additionally, this is the first study to compare the vaccine response to SARS-CoV-2 among different forms of systemic vasculitis, while other studies were restricted to include only specific forms of vasculitis (45–51) or compared the immunogenicity of vasculitis with other IMDRs (95–97). In our study, patients with AAV, TAK, and BD demonstrated equivalent vaccine responses, which contrasts with most AAV series reporting lower seroconversion rates than other diseases (45, 47, 49, 51). The seropositivity rates in our AAV patients reached 69.2%, 84.2%, and 100% after each dose of SARS-CoV-2 vaccine, whereas other studies reported 0–21% after the first dose and 28–66% after the second dose (45, 47, 49, 51). We attribute this difference to the low frequency of rituximab use in our cohort. These findings reinforce the idea that impaired humoral responses in AAV are largely driven by RTX exposure rather than by the underlying disease itself (45, 46, 48, 49).

Our study is the first to evaluate the immunogenicity against SARS-CoV-2 in patients with TAK (61). We have shown clinical effectiveness, safety, and a low rate of disease relapse in this population. In a previous study, patients with TAK were included as a whole group in a large cohort of systemic autoimmune diseases, but no analyses were made regarding their vaccine response (98). Other studies have evaluated the behavior of TAK patients during the COVID-19 pandemic, and reported flare rates of 28.5%. These flares were most often associated with unsupervised discontinuation of immunosuppressive therapy or delays in medical follow-up (60). In contrast, a Chinese study found that a SARS-CoV-2 infection itself did not increase the risk of flare, with relapse rates actually lower among infected compared with uninfected patients (28). European surveys reported that most TAK patients were in remission, and no flares were attributed to either infection or vaccination (99, 100).

Furthermore, in cohort studies including different IMDR, the vasculitis group had a reduced response compared to other diseases, usually at the expense of AAV patients using RTX (95–97). In another study that compared vasculitis patients from the SAFER study with other systemic autoimmune diseases in the same cohort, there was a trend toward a lower humoral response in patients with vasculitis, inflammatory myopathies, and systemic sclerosis, while patients with Sjögren’s disease and systemic lupus erythematosus tended to have a better response to SARS-CoV-2 vaccination (42).

Since this was a planned vaccination study, most patients were in remission at baseline, and this profile was maintained throughout the follow-up. The frequency of active disease remained stable in the follow-up visits of the study. Active disease was found in 4.6% of the whole group and in 16.0%, 8.6% and 9.0% in AAV, BD and TAK patients, respectively. Additionally, the median scores for disease activity, such as BVASv3 and BR-BDCAFs were at similar levels throughout the study, reinforcing that the vaccine did not increase disease activity scores or relapses in patients in remission or with mild disease activity.

One patient with EGPA developed a severe disease relapse with a significant temporal relation after the vaccine. In this case, the disease activity was already underway at the time of the third vaccine dose and we believe that the vaccine likely influenced the worsening of the disease. Indeed, there is a theoretical risk of autoimmune disease exacerbation after COVID-19 vaccination, although severe relapses are rare, and the benefits of vaccination outweigh these risks. In this context, the American College of Rheumatology (ACR) guidelines recommend that vaccination can be administered to patients with active disease, as long as it is not severe, whereas patients presenting life-threatening manifestations or major organ dysfunction should wait for disease control before immunization (101).

Other studies corroborate the safety of SARS-CoV-2 vaccination concerning disease relapses in systemic vasculitis, as most of them showed low disease relapse rates after vaccination. A cohort with similar features to ours, including different types of systemic vasculitis, observed a 0.9% rate of disease relapses in 107 patients (102). In patients with AAV, no increased frequency of relapses or hospitalizations were observed after SARS-CoV-2 vaccination (45, 64). Another study also found a low frequency of relapses (i.e., 0.5%) in patients with IgAV, characterized by transient renal function impairment, but with no severe adverse events (63). Disease relapses were observed in 5.3% to 12.7% of patients with cryoglobulinemic vasculitis (61, 62) and in 7.1% of patients with GCA who underwent SARS-CoV-2 vaccination (58). Regarding SARS-CoV-2 vaccination in BD, studies conducted in Turkey reported higher relapse rates in BD (i.e., 16% to 53%) compared to our findings (54, 55). Relapses were mostly mild and mucocutaneous. However, up to 4% of BD patients experienced severe relapses, including uveitis, venous thrombosis, and even neurological involvement (54). Notably, Turkey is an endemic country for BD, providing larger and more representative samples for such studies. In relation to SARS-CoV-2 vaccination in TAK, an internet-based data collection revealed results comparable to ours, showing a low rate of disease relapse (i.e., 8%) by TAK patients after vaccination (28). These data indicate that the frequency and susceptibility to relapses upon SARS-CoV-2 vaccination may vary among different types of vasculitis. However, the overall relapse rates are low and comparable to the usual disease activity rates in this population, which reinforces the safety of SARS-CoV-2 vaccination for these patients.

The profile of AEs observed in this study was predominantly mild. Injection-site pain, fatigue, and headache were the most frequent AEs, which is similar to the literature (45, 47, 54, 55, 57, 62, 102). Additionally, age appears to be a protective factor for the frequency of AEs seemed to be lower in older compared to younger patients with vasculitis (57, 102).

### 4.1 Limitations

We acknowledge some limitations to this study. The main limitation is the small sample size, which, combined with the heterogeneity of vasculitis subtypes and concomitant medications, may have limited the power to perform specific analyses, such as the impact of immunosuppressive therapies on vaccine response. Consequently, the study is underpowered for such analyses. However, it provides valuable real-life data that, when combined with findings from other cohorts, may help generate more consistent evidence. Another limitation was the loss of patients during follow-up, although the patient loss in our study was similar to that reported in other studies (55). Additionally, the difficulty in monitoring and confirming COVID-19 cases was challenging, as PCR testing was not performed in all suspected cases, and over time, people ceased testing. To minimize underreporting, we included unconfirmed typical COVID-19 cases (e.g., anosmia and known contact with confirmed COVID-19 cases) and we focused on severe COVID-19 presentations. Finally, the absence of a non-vasculitis control group limits direct comparisons with the general population.

### 4.2 Clinical implications and future directions

Despite these limitations, our study provides novel real-life data on SARS-CoV-2 vaccination in systemic vasculitis, which is a rare and clinically diverse group of diseases. We directly compared vaccine responses across vasculitis subtypes and provided longitudinal data on TAK. Clinically, the results reinforce current recommendations to complete at least three vaccine doses to achieve adequate immunogenicity, as well as to prioritize vaccination for patients in remission, with careful timing in relation to immunobiological therapies such as RTX (103). The observed reassuring safety profile may also contribute to reducing vaccine hesitancy among patients and physicians. Although limited, the study provides valuable real-life data on rare diseases. Additionally, combining our findings with results from other cohorts may generate more consistent evidence (48, 49, 53, 58, 61, 103).

## 5 Conclusion

To the best of our knowledge, this is the first real-life study to compare the immunogenicity, clinical efficacy, and safety of different SARS-CoV-2 vaccines in a population comprising exclusively patients with systemic vasculitis, including the first longitudinal data reported in TAK. Our study emphasizes the importance of completing a minimum of three vaccine doses to ensure adequate immunogenicity and that vaccination is safe in patients with low disease activity or in remission, with no increased risk of relapse. Hence, our results support the recommendations to prioritize vaccination for patients in remission and to time vaccinations according to the nadir of immunobiological drugs, such as RTX.

## Data Availability

The raw data supporting the conclusions of this article will be made available by the authors, without undue reservation.
