# Supplementary material for: Immunogenicity and safety to SARS-Cov-2 vaccination in patients with systemic vasculitis
Source: Front Immunol. 2025 Nov 17;16:1655917. doi: 10.3389/fimmu.2025.1655917 (PMC12665670; doi:10.3389/fimmu.2025.1655917)
Supplement: Supplementary file 1 [file DataSheet1.pdf]

Supplementary Material

Supplementary Table S1 – Comparison between drug therapy and vaccines among the main groups of systemic vasculitis at baseline.

Variables	Total (n=59)	Behçet's disease (n=30)	Takayasu arteritis (n=15)	AAV (n=14)	*p*
			Therapeutic agents		
TNFi or Tocilizumab, %	24/59 (40.6)	14/30 (46.6)	10/15 (66.6)	0/14 (0.0)	<0.001*
Rituximab, %	3/59 (5.0)	0/30 (0.0)	0/15 (0.0)	3/14 (21.4)	0.011*
csDMARD, %	18/59 (30.5)	12/30 (40.0)	2/15 (13.3)	4/14 (28.5)	0.18
MMF, %	8/59 (13.5)	2/30 (6.6)	4/15 (26.6)	2/14 (14.2)	0.20
MTX, %	10/59 (16.9)	3/30 (10.0)	3/15 (20.0)	4/14 (28.5)	0.24
>20mg/week	4/10 (40.0)	0/3 (0.0)	2/3 (66.6)	2/4 (50.0)	NA
Daily prednisone or equivalent, %	22/59 (37.2)	9/30 (30.0)	8/15 (53.3)	5/14 (35.7)	0.32
> 20mg	4/22 (18.18)	0/9 (0.0)	1/8 (12.5)	3/5 (60.0)	NA
			Vaccines		
CoronaVac	25/59 (42.3)	9/24 (37.5)	5/11 (45.4)	6/14 (42.8)	0.96
ChAdOx1	38/59 (16.9)	15/24 (62.5)	7/11 (63.6)	8/14 (57.2)	0.98
			Vaccination schemes		
Homologous	40/59 (68.8)	10/26 (38.5)	3/11 (27.3)	1/12 (7.5)	0.32
Heterologous	19/59 (32.2)	16/26 (61.5)	8/11 (72.7)	11/12 (91.6)	0.74

AAV – Antineutrophil cytoplasmic antibodies associated vasculitis; csDMARD – Conventional synthetic disease modifying antirheumatic drugs; MMF – Mycophenolate mofetil; MTX – Methotrexate; n – Number of patients; NA – Not applicable; TNFi – Tumor necrosis factor inhibitors; * - Flags significant results.

Supplementary Table S2 – Comparisons of IgG-RBD titers between vasculitis patients undergoing different SARS-CoV-2 vaccination schemes.

**Time points**	**Groups of SARSCoV-2 vaccination**			***p***
	SARS-CoV-2 Vaccines			
	CoronaVac (n=25)	ChAdOx1 nCoV-19 (n=36)		
T0	2.10 (2.20)	1.01 (1.50)		0.027*
T1	3.44 (2.10)	4.23 (2.36)		0.20
T2	4.06 (2.08)	5.97 (1.94)		0.001*
T3	6.45 (2.00)	6.64 (1.46)		0.71
	Vaccination schemes			
	Heterologous scheme (n=40)	Homologous scheme (n=19)		
T0	1.62 (2.16)	1.19 (1.81)		0.46
T1	4.04 (2.45)	3.86 (2.24)		0.82
T2	5.44 (2.20)	5.16 (2.20)		0.66
T3	7.01 (1.58)	6.14 (1.52)		0.073
	Vasculitis			
	Behçet's disease (n=30)	Takayasu arteritis (n=15)	AAV (n=14)	
T0	1.15 (2.01)	1.62 (1.40)	1.48 (2.08)	0.70
T1	3.79 (2.39)	4.28 (2.12)	3.70 (2.38)	0.80
T2	5.35 (2.07)	5.31 (1.99)	5.15 (2.70)	0.96
T3	6.93 (1.51)	6.54 (1.85)	6.50 (1.62)	0.70

Results are presented as mean (standard deviation) of the log of IgG-RBD BAU; AAV – ANCA-associated vasculitis; BAU – Binding antibody units; n – Number of patients; RBD – Receptor binding domain; T0: Baseline; T1: 28 days after the 1st dose; T2: 28 days after the 2nd dose; T3: 28 days or more after the 3rd dose;* - Flags significant results.

Supplementary Table S3 – Seropositivity against SARS-CoV-2 after Coronavac or ChAdOx1 nCoV-19, and after heterologous or homologous vaccine regimens.

	Total<br>N=61	CoronaVac<br>N=25	ChAdOx1<br>N=36	*P*	Total<br>N=59	Heterologous<br>N=40	Homologue<br>N=19	*P*
Seropositivity at baseline, %	16/59 (27.12)	11/23 (47.83)	5/36 (13.89)	**0.004**	14/58 (24.14)	12/39 (30.77)	2/19 (10.53)	0.11
Seropositivity after 1st dose, %	44/56 (78.57)	17/23 (73.91)	27/33 (81.82)	0.48	38/49 (77.55)	28/36 (77.78)	10/13 (76.92)	0.95
Seropositivity after 2nd dose, %	49/55 (89.09)	17/21 (80.95)	32/34 (94.12)	0.13	50/55 (90.91)	35/38 (92.11)	15/17 (88.24)	0.64
Seropositivity after 3rd dose, %	48/49 (97.96)	16/17 (94.12)	32/32 (100.00)	0.17	50/51 (98.04)	34/35 (97.14)	16/16 (100.00)	0.49

Supplementary Table S4 – Seropositivity against SARS-CoV-2 between different forms of vasculitis

	Total<br>N=59	Behçet<br>N=30	Takayasu<br>N=15	ANCA<br>N=14	*P*
Seropositivity at baseline, %	15/58 (25.86)	5/30 (16.67)	6/15 (40.00)	4/13 (30.77)	0.22
Seropositivity after 1st dose, %	37/47 (78.72)	17/22 (77.27)	11/12 (91.67)	9/13 (69.23)	0.38
Seropositivity after 2nd dose, %	45/50 (90.00)	24/26 (92.31)	10/11 (90.91)	11/13 (84.62)	0.75
Seropositivity after 3rd dose, %	43/44 (97.73)	22/23 (95.65)	10/10 (100.00)	11/11 (100.00)	0.63

Supplementary Table S5 – Frequency of suspected and/or confirmed COVID-19 cases after the 1st and 2nd doses of CoronaVac and ChAdOx-1 vaccines.

**Variables**	**Total** (n=61)	**CoronaVac** (n=25)	**ChAdOx-1** (n=36)	***p***
Suspected/positive cases at T1, n (%)	3/61 (4.9)	3/25 (12.0)	0/36 (0.0)	0.064
Suspected/positive cases at T2, n (%)	8/61 (13.1)	2/25 (8.0)	6/36 (13.9)	0.45

T1: 28 days after the first dose; T2: 28 days after the second dose of the SARS-CoV-2 vaccine.

Supplementary Table S6 - Frequency of suspected and/or confirmed COVID cases after the 3rd dose in homologous and heterologous vaccination schemes.

Variable	Total (n=59)	Heterologous scheme (n=40)	Homologous scheme (n=19)	*p*
Suspected/positive cases at T3, n (%)	10/59 (16.9)	4/40 (10.0)	6/19 (26.3)	0.062

T3: 28 days after the third dose of the SARS-CoV-2 vaccine.

Supplementary Table S7 – Comparisons of safety between ChAdOx1 nCoV-19 and BNT162b2 vaccines in vasculitis patients after the booster dose.

**Adverse events**	**ChAdOx1 nCoV-19 (n=15)**	**BNT162b2 (n=45)**	***p***
Up to 28 days after the third dose			
Site-injection pain, n (%)	7/15 (46.7)	24/34 (70.6)	0.11
Skin rashes, n (%)	1/15 (6.7)	0/34 (0.0)	0.31
Nausea or vomiting, n (%)	0/1 (0.0)	--	--
Fatigue, n (%)	4/15 (26.7)	6/34 (17.7)	0.47
Headache, n (%)	5/15 (33.3)	8/34 (23.5)	0.50
Myalgia, n (%)	5/15 (33.3)	9/34 (26.5)	0.73
Arthralgia, n (%)	4/15 (26.7)	7/34 (20.6)	0.72
Fever, n (%)	4/15 (26.7)	9/34 (26.5)	1.00
Dizziness, n (%)	2/15 (13.3)	6/34 (17.7)	1.00

n – Number of patients; * - Flags significant results.
